# *De Novo* Assembly and Transcriptome Analysis of Contrasting Sugarcane Varieties

**DOI:** 10.1371/journal.pone.0088462

**Published:** 2014-02-11

**Authors:** Claudio Benicio Cardoso-Silva, Estela Araujo Costa, Melina Cristina Mancini, Thiago Willian Almeida Balsalobre, Lucas Eduardo Costa Canesin, Luciana Rossini Pinto, Monalisa Sampaio Carneiro, Antonio Augusto Franco Garcia, Anete Pereira de Souza, Renato Vicentini

**Affiliations:** 1 Center for Molecular Biology and Genetic Engineering (CBMEG), University of Campinas (UNICAMP), Campinas, SP, Brazil; 2 Centro Avançado da Pesquisa Tecnológica do Agronegócio de Cana (IAC/Apta), Ribeirão Preto, SP, Brazil; 3 Departamento de Biotecnologia e Produção Vegetal e Animal, Centro de Ciências Agrárias, Universidade Federal de São Carlos, Araras, SP, Brazil; 4 Departamento de Genética, Escola Superior de Agricultura Luiz de Queiroz, Universidade de São Paulo, Piracicaba, SP, Brazil; 5 Departamento de Biologia Vegetal, Instituto de Biologia, Universidade Estadual de Campinas (UNICAMP), Campinas, SP, Brazil; University of North Carolina at Charlotte, United States of America

## Abstract

Sugarcane is an important crop and a major source of sugar and alcohol. In this study, we performed *de novo* assembly and transcriptome annotation for six sugarcane genotypes involved in bi-parental crosses. The *de novo* assembly of the sugarcane transcriptome was performed using short reads generated using the Illumina RNA-Seq platform. We produced more than 400 million reads, which were assembled into 72,269 unigenes. Based on a similarity search, the unigenes showed significant similarity to more than 28,788 sorghum proteins, including a set of 5,272 unigenes that are not present in the public sugarcane EST databases; many of these unigenes are likely putative undescribed sugarcane genes. From this collection of unigenes, a large number of molecular markers were identified, including 5,106 simple sequence repeats (SSRs) and 708,125 single-nucleotide polymorphisms (SNPs). This new dataset will be a useful resource for future genetic and genomic studies in this species.

## Background

Sugarcane belongs to the grass family (Poaceae), which is an economically important seed plant family that includes maize, wheat, rice, sorghum and many types of grasses. The sugarcane crop is the main source of both sugar and alcohol, accounting for two-thirds of the world's sugar production [1]. It is estimated that approximately 653.81 million tons of sugarcane will be produced during the 2013/2014 harvest in Brazil, surpassing the production of the last harvest [2].

Modern sugarcane varieties are derived from interspecific hybridization between *Saccharum officinarum* and *Saccharum spontaneum*, resulting in highly polyploid and aneuploid plants. Indeed, the chromosome number of these varieties ranges from 80 to 140. Modern varieties of sugarcane typically exhibit more than eight homologous copies of each basic chromosome from *S. officinarum* and several copies of the homologous chromosomes from *S. spontaneum* [3]. Therefore, sugarcane cultivars are highly heterozygous, presenting several different alleles at each locus, and this high level of genetic complexity creates challenges during conventional and molecular breeding programs.

Recent technological developments have the potential to greatly increase our understanding of sugarcane plants through the application of emerging genomic technologies, and the use of next-generation sequencing (NGS) technologies could have significant implications for crop genetics and breeding. Although the sequencing of large genomes remains expensive, even using NGS technologies [4], transcriptome sequencing can provide information regarding the gene content of a species and can complement genome sequencing approaches.

RNA sequencing (RNA-Seq) has been applied as a tool for transcriptome analysis in many species, such as *Arabidopsis thaliana* [5], *Brassica* spp. [6], rice [7] and maize [8]. RNA-Seq has several advantages, including (i) allowing more precise measurement of the levels of transcripts and their isoforms than other methods, (ii) presenting the potential for the development of SNPs that can be used to detect allele-specific expression because the same base is sequenced multiple times, (iii) the ability to identify reads containing post-transcriptional modifications or rearranged sequences that cannot be mapped directly to the genome [9] and (iv) allowing the identification of species-specific genes [10]. Moreover, the availability of a large number of genetic markers developed using NGS technologies is facilitating trait mapping and marker-assisted breeding [11].

In plant breeding programs, genotypes of interest to breeders, such as the parental genotypes of mapping populations, can be sequenced using NGS technologies. More than one genotype can be employed to generate sequence data with these technologies, and these data can be aligned using genome or transcriptome sequencing data for model or major crop species that are closely related to the species of interest [11]. This approach has also been applied for marker discovery in some crop species, such as eucalyptus [12], maize [13] and chickpea [14], and has been used to identify SNPs between the parental genotypes of mapping populations. These SNPs can then be employed to develop markers for marker-deficient crops to allow trait mapping through marker-assisted selection (MAS).

Despite its economic importance, no published genome sequence is currently available for sugarcane. Instead, the basic resource used for the study of sugarcane gene sequences is the substantial expressed sequence tag (EST) information available in public databases. Transcriptome studies in sugarcane began in South Africa [15], [16], and the largest EST collection (∼238,000 ESTs) was developed through the Brazilian SUCEST project [17], [18]. Researchers in Australia [19]–[21] and the USA [22] have generated three additional libraries containing 10,000 ESTs each. Currently, all of the reported ESTs are collected in the Sugarcane Gene Index, version 3.0, which contains 282,683 ESTs and 499 complete cDNA sequences, resulting in 121,342 unique assembled sequences, or unigenes. There are still more than 10,000 sugarcane coding genes that have yet to be identified [23], highlighting the need for new sequencing efforts in the sugarcane transcriptome. This information would increase the panel of potential molecular markers and sequence information available for sugarcane breeding programs, resulting in biotechnological improvements. In the present study, using the Illumina GA IIx sequencing platform, we performed *de novo* transcriptome sequencing in six sugarcane genotypes that are employed as parents in Brazilian Sugarcane Breeding Programs. We identified conserved genes that have not previously been described in sugarcane, and these data will be useful for future genome assembly and marker identification.

## Materials and Methods

### Ethics Statement

We confirm that no specific permits were required for the described field studies. This work was a collaborative research project developed by researchers from UNICAMP, ESALQ/USP, IAC/Apta (Instituto Agronômico de Campinas) and UFSCar-RIDESA (Universidade Federal de São Carlos-Rede Interinstitucional de Desenvolvimento do Setor Sucroalcooleiro) (all from Brazil). We also confirm that the field studies did not involve endangered or protected species.

### Plant Materials and RNA Extraction

Six genotypes were included in this study. IACSP96-3046 and IACSP95-3018 are the parents of a mapping population from the Sugarcane Breeding Program at IAC/Apta. IACSP95-3018 is a promising clone that is also used as a parent in the breeding program. IACSP93-3046 is a variety that exhibits good tillering, an erect stool habit [24] and resistance to rust [25].

SP81-3250×RB925345 and SP80-3280×RB835486 are the parents of two different mapping populations from the Sugarcane Breeding Program at UFSCar, which is part of RIDESA. These parents exhibit contrasting properties: SP81-3250 and SP80-3280 are resistant to rust [26], [27], whereas RB925345 and RB835486 are susceptible [28]. All of the examined genotypes display high levels of sucrose.

Leaves at the third position [29] were collected from one plant per genotype and immediately frozen, and total RNA was extracted using a modified protocol [30]. The integrity and quantity of the isolated RNA were assessed using a 2100 Bioanalyzer (Agilent). Equal quantities of high-quality RNA from each genotype were pooled for cDNA synthesis.

### mRNA-Seq Library Construction for Illumina Sequencing

Paired-end Illumina mRNA libraries were generated from 4 μg of total RNA in accordance with the manufacturer's instructions for mRNA-Seq Sample Preparation (Illumina Inc., San Diego, CA, USA). The quality of the library was assessed using a 2100 Bioanalyzer (Agilent Technologies, Palo Alto, CA, USA).

Cluster amplification was performed using the TruSeq PE Cluster Kit and a cBot (Illumina), and each sample was sequenced in a separate GAIIx lane using the TruSeq SBS 36 Cycle Kit (Illumina). The read length was 72 bp.

### Sequence Data Analysis and Assembly

The raw data generated by Illumina sequencing were converted from the BCL format to qSeq using Off-line Basecaller, v.1.9.4 (OLB) software. The qSeq files were transformed in FastQ files, which contain sequences that are 72 bp in length, using a custom script. Low-quality sequences were removed; these sequences included reads with ambiguous bases, reads with less than 70 bases, and reads with a Phred quality score Q≤20 using the NGS QC toolkit [31]. All reads were deposited in the National Center for Biotechnology Information (NCBI) database and can be found under accession number SRA073690.

All datasets were combined, and the sequenced reads were assembled using Trinity (http://trinityrnaseq.sourceforge.net/), which is a program developed specifically for *de novo* transcriptome assembly from short-read RNA-Seq data that recovers transcript isoforms efficiently and sensitively using the de Bruijn graph algorithm [32]. The optimal assembly results were chosen according to an evaluation of the assembly encompassing the total number of contigs, the distribution of contig lengths, the N50 statistic and the average coverage. The assembled transcripts were based on the main isoform of each transcript, and only contigs with lengths of greater than 300 bp were included in the downstream analysis.

To identify the genotypic contribution to each transcript, reads from each library were mapped against the assembly generated from all libraries using the bowtie aligner [33]. The BAM files generated by bowtie were then used to estimate the transcript-level abundance for each library using the RSEM (RNA-Seq by Expectation Maximization) software [34].

### Functional Annotation of Sugarcane Transcripts

The assembled sequences were compared against the NCBI non-redundant protein database (NR) using BLASTX with a cut-off E-value of 10^-6^. To annotate the assembled sequences according to Gene Ontology (GO) terms (The Gene Ontology Consortium, 2000), the above BLAST results were analyzed using Blast2GO [35] to determine and compare gene functions. The GO terms were assigned to the representative transcripts for each sample through an enrichment analysis using Fisher's exact test (p-value <0.01), with a false discovery rate (FDR) correction in terms of biological processes and molecular functions. The transcript sequences were also aligned against the *Viridiplantae*, grass and sorghum protein databases (http://www.phytozome.org/) using BLASTX and against the Sugarcane Gene Index (http://compbio.dfci.harvard.edu/tgi/) using BLASTN; in both alignments, a cut-off E-value of 10^−6^ was applied. The BLAST search was limited to the first ten significant query hits, and the gene names were assigned to each query based on the highest score. Transcripts that showed similarity to *Viridiplantae* proteins were aligned against the sorghum genome using sim4 software [36]. Open reading frames (ORFs) were predicted using a script available in the TransDecoder package (http://transdecoder.sourceforge.net/), with 300 bp as the minimum ORF length. Those transcripts showing predicted ORFs were aligned against grass proteins using the STRING database, v.9.05 (http://string-db.org), to predict Clusters of Orthologous Groups (COG).

To further characterize the subset of unigenes that did not show similarity to any known plant proteins, we applied a computational strategy to mine putative long non-coding RNA (lncRNA) data. We first aligned all 121,342 EST unigenes to *Viridiplantae* proteins and to the GenBank NR database using BLASTX. Those EST unigenes that did not align with any proteins were then mapped to the *Sorghum bicolor* genome, obtaining at least 70% coverage and a maximum intron size of 15 kb. The coding probability of the positively mapped unigenes was then evaluated by removing sequences with potential ORFs longer than 100 aa using ESTScan [37]. We further investigated the functional role of the remaining unigenes and putative lncRNAs by searching for three indirect indications of functionality: we examined the stability of the secondary structure using the Vienna package [38], normalized to the Z-score index [39]; we mapped the small RNAs (sRNAs) [40] against sugarcane unigenes; and we analyzed the sequence similarities between the unigenes and *S. bicolor* ESTs (BLASTN, E-value ≤1e^−5^). Only EST unigenes with at least one indirect piece of functional evidence were analyzed further. The putative lncRNAs were then aligned to the 18,910 assembled transcripts that showed no similarity to any plant protein but were successfully mapped to *S. bicolor* (Text S4). Only hits with an E-value below 1e^−5^ and coverage higher than 40% were considered positive.

### Putative Molecular Markers

We utilized the MISA program (http://pgrc.ipk-gatersleben.de/misa/) to search for simple sequence repeat (SSR) motifs in the unigenes; the MISA script can identify both perfect and compound (interrupted by a certain number of bases) motifs. To identify the presence of SSRs, only motifs of two to six nucleotides were considered, and the minimum repeat unit was defined as six for dinucleotide motifs and five for tri-, tetra-, penta- and hexanucleotide motifs. A compound motif was defined as two or more SSR motifs interrupted by sequences of up to 100 bp.

To identify putative single-nucleotide polymorphisms (SNPs) in the sugarcane transcript assembly, we first separately mapped all of the short reads from each library to the assembly using the Burrows-Wheeler Aligner (BWA). Next, FreeBayes [41] and SAMtools [42] were used to detect the variable positions of SNPs from the consensus sugarcane assembly. The FreeBayes tool allowed us to identify genetic variants in the polyploid organisms. The putative SNPs were then filtered using the varFilter command, where variants were called only for positions with a minimal mapping quality (-Q) and coverage (-d) of 25. To compare the composition of the SNP variation in the parental genotype, unique and shared SNPs were extracted using an in-house script. The transition and transversion ratios were calculated using the tstv tool developed by SnpSift software [43].

## Results and Discussion

### *De novo* assembly of the sugarcane transcriptome

The libraries sequenced using the Illumina platform produced a total of 610,232,490 paired-end (PE) sequence reads, each of which was 72 bp in length. We filtered the sequence data for low-quality reads, resulting in 445,374,504 high-quality PE trimmed reads (97.67%), which were used to obtain the *de novo* assembly. An overview of the sequencing procedure is presented in Table 1. The *de novo* assembly generated 119,768 transcripts when all isoforms were considered. These transcripts represent a total of 72,269 unigenes that were considered for downstream analysis (Text S1). The length of the unigenes ranged from 300 bp to ∼7 kb, with a mean length of 921 bp, an N50 equal to 1,367 bp and 46.39% GC content. The average length of the assembled unigenes was greater than those obtained from chickpea (523 bp) [14], rubber trees (485 bp) [44] and bamboo (736 bp) [45] using similar sequencing technologies. Considering the N50 values, the values for the sugarcane unigenes were greater than those for rubber trees (592 bp), bamboo (1,132 bp) and chili pepper (1,076 bp) [46], which were also assembled using short reads generated by the Illumina platform. In total, we obtained 18,624 (27.21%) unigenes longer than 1 kb and 7,657 (10.6%) unigenes longer than 2 kb. The length distributions of the unigenes are shown in Table 2, revealing that more than 40,000 unigenes (55.76%) were longer than 500 bp. These unigenes were submitted to an ORF predictor using TransDecoder, and we detected 33,673 (46.59%) unigenes with ORFs, with 9,350 (12.94%) presenting complete ORFs.

**Table 1:** Summary of Illumina transcriptome sequencing data for the sugarcane varieties included in this study.

Sample	Read length (bp)	Raw data	Trimmed data	GC (%)	Q20 (%)
**SP95-3018**	72+72	84,105,462	64,906,391	49.04	98.09
**SP81-3250**	72+72	103,971,718	71,002,186	47.52	97.32
**RB925345**	72+72	112,124,334	77,476,268	46.91	97.11
**SP80-3280**	72+72	101,983,186	73,160,814	47.59	97.56
**RB835486**	72+72	119,280,444	87,873,521	46.62	97.66
**SP93-3046**	72+72	88,767,346	70,955,324	48.07	98.25

**Table 2:** Summary of the *de novo* assembly results for the sugarcane transcriptome.

Unigene length (bp)	Total unigenes	Percentage
300–500	31,971	44.24%
500–1000	20,634	28.55%
1000–2000	12,007	16.61%
2000–3000	4,827	6.68%
3000–4000	1,790	2.47%
4000–5000	636	0.88%
>5000	404	0.56%
Total length (bp)	66,572,642	-
Unigenes	72,269	-
N50 length	1,367	-
GC (%)	46.39	-

### Unigene annotation

**Figure 1:**
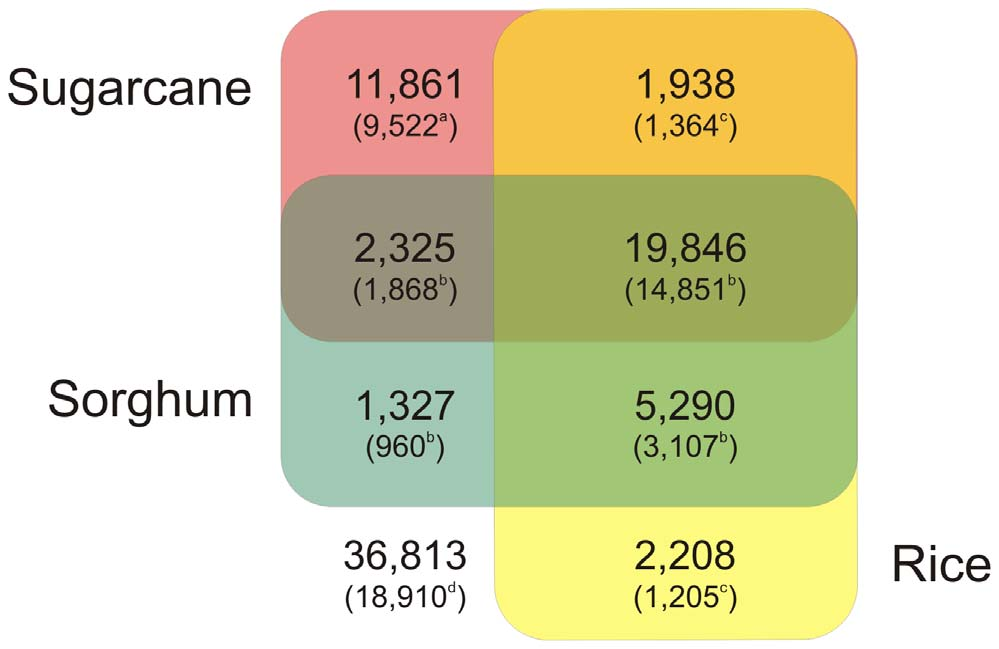
Proportions of sugarcane transcripts showing homology to sugarcane unigenes and sorghum and rice proteins. For annotation, the best BLASTX/N hit against the protein or nucleotide sequences of the reference organisms was employed, with an E-value cut-off of ≤10^−6^. The number between the parentheses indicates the number of different proteins/unigenes in each species (sugarcane^a^, sorghum^b^ and rice^c^). The number outside of the Venn diagram indicates no-hit transcripts and the number of transcripts^d^ that mapped to the sorghum genome.

The 72,269 sugarcane unigenes were analyzed for sequence similarity against the *Viridiplantae* (comprising all green plants) and grass (*S. bicolor, Oryza sativa, Zea mays, Panicum virgatum, Setaria italica* and *Brachypodium virgatum*) datasets through BLASTX searches. The unigenes were also compared against the sugarcane EST database via a BLASTN search (Table 3). A total of 35,456 (49.06%) unigenes showed significant similarity to *Viridiplantae*. The high percentage of sugarcane unigenes obtained in this study that did not match the *Viridiplantae* protein database (50.84%) indicates that there is potential for the discovery of as-yet-undescribed and novel genes in sugarcane, although most of these unigenes may encode non-coding RNAs. In fact, more than 26% of the unigenes in this set exhibited high similarity to intergenic regions of the sorghum genome (Figure 1). Additionally, the significance of a BLAST search depends on the length of the query sequence; therefore, short sequences are rarely matched to known genes [12], or these sequences may represent rapidly evolving sequences that have diverged substantially from their homologs [47].

**Table 3:** Summary of the annotation of each database.

Database	Number of unigenes	Number of proteins matched	Percentage of unigenes^a^
Viridiplantae proteins	35,456	34,969	49.06%
Grass proteins	34,814	34,304	48.17%
Sorghum proteins	28,788	28,030	39.83%
Hits against sorghum proteins and sugarcane ESTs	22,171	20,969	30.68%
Total of no-hit unigenes	36,813	-	50.94%
No-hit unigenes with high similarity to the sorghum genome	18,910	-	26,16

a Percentage relative to the total number of sugarcane unigenes.

In turn, alignment of the unigenes against the grass protein database returned 34,814 significant hits. When considering the hits by species, 28,788 unigenes showed significant similarity to sorghum, corresponding to 98% of sorghum proteins (Figure 1). These results were expected, as comparative genomic studies [48] have revealed conservation and synteny among the sugarcane and sorghum genomes. The sugarcane transcriptome also significantly matched that of rice, with approximately 29,285 unigenes (corresponding to 28,732 unique protein accessions) showing significant similarity to rice proteins.

To investigate previously unidentified potential genes in sugarcane, we compared the unigenes against the sugarcane transcripts deposited in public databases and performed BLAST searches to detect possible similarities with the SoGI database (*S. officinarum*). Furthermore, the unigenes that did not show similarity to sugarcane ESTs were compared against sorghum proteins. Approximately 22,171 unigenes exhibited significant similarity to sorghum proteins and sugarcane transcripts (Figure 1). The remaining 5,272 unigenes (Text S3) showed significant similarity to sorghum and rice proteins but not to the sugarcane transcripts that were considered to be putative new sugarcane genes (Figure 1). By examining the presence of candidate coding regions in these unigenes, we identified 4,895 sequences that contained ORFs, with 732 unigenes containing complete ORFs. These unigenes represent genes that have not yet been described for sugarcane.

### Clusters of Orthologous Groups (COG) classification

**Figure 2:**
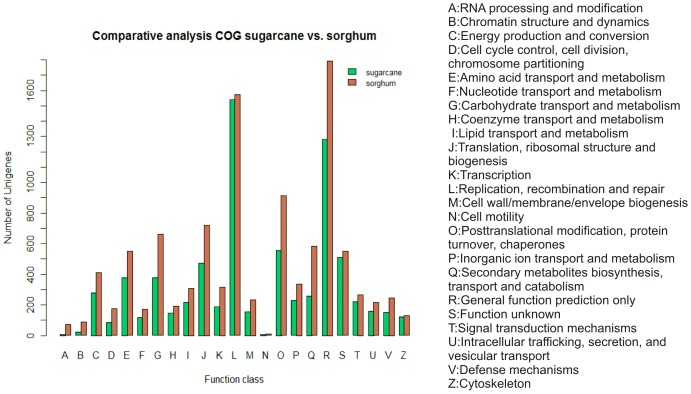
Histogram of the Clusters of Orthologous Groups (COG) classifications of the sugarcane transcripts and sorghum proteins.

COG classification was performed for the transcriptome data, and a total of 7,519 unigenes were identified (Figure 2). These unigenes were classified into 23 COG categories, with the largest number of unigenes being grouped in the ‘replication, recombination and repair’ cluster (20.49%), followed by the ‘general function prediction only’ cluster (17.05%) and the ‘posttranslational modification, protein turnover and chaperones’ cluster (7.39%). These three categories are the same categories that are highly represented in sorghum (Figure 2).

A total of 19 of the 23 COG categories were present in the transcriptome data, and at least 60% of the sugarcane unigenes were annotated when compared with the annotation of sorghum genes in the COG categories.

The categories ‘energy production and conversion’ (3.72%), ‘carbohydrate transport and metabolism’ (5%) and ‘defense mechanisms’ (2%) exhibited at least 56% of the expected genes compared with the sorghum genes. These categories should be considered to represent gene sequences showing a high potential for the development of molecular markers in sugarcane breeding programs. Therefore, the likelihood of these markers being associated with agronomic traits of interest in QTL mapping and marker-assisted selection (MAS) [49] is increased.

### Gene Ontology enrichment analyses

**Figure 3:**
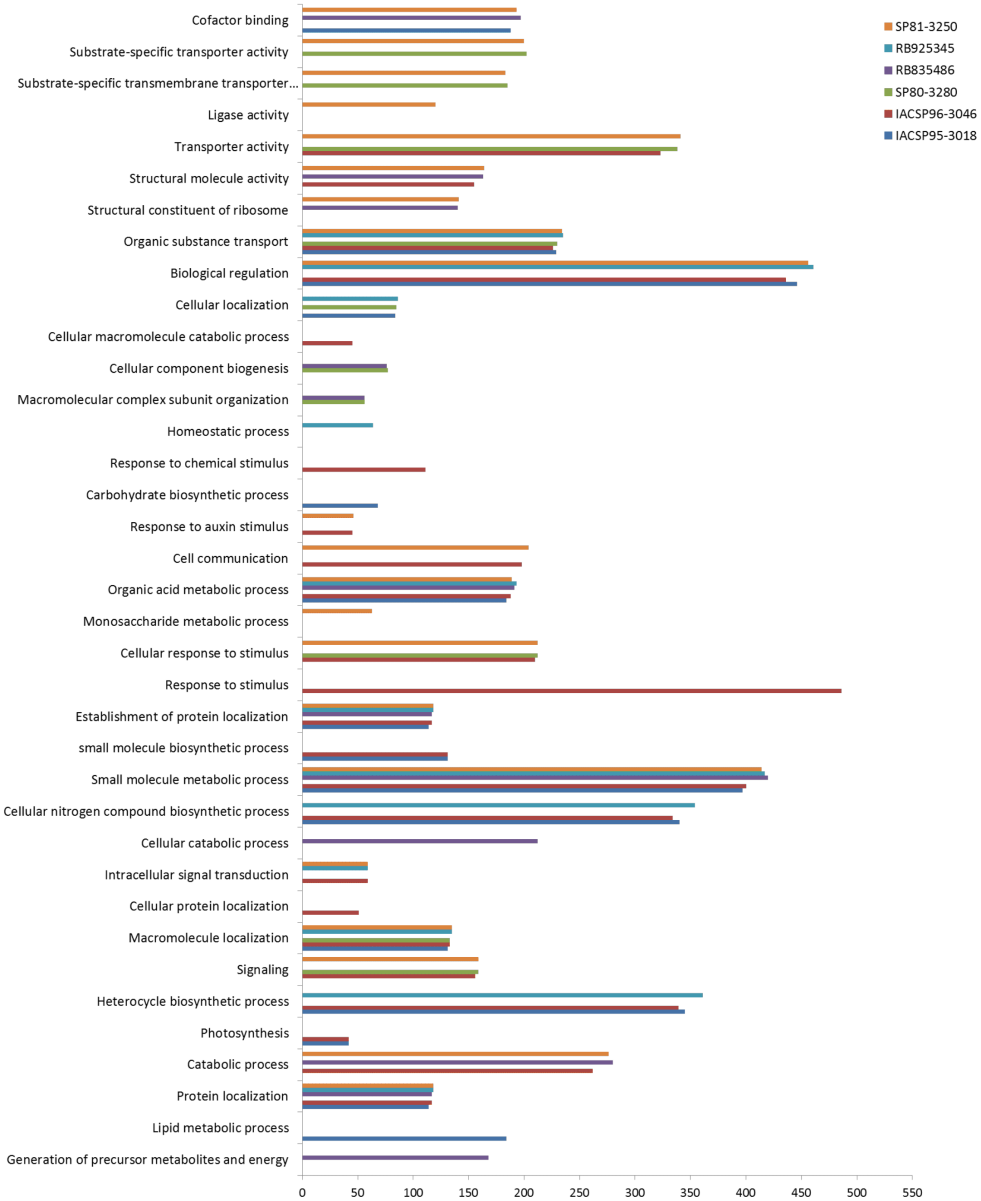
Enrichment of Gene Ontology terms for each sugarcane variety.

The identification of functional classes that differ statistically between two lists of terms is a typical data-mining approach applied in functional genomics research [35]. In this work, we were interested in identifying which functions were distinctly represented among the different sugarcane genotypes. A total of 14,983 unigenes (Text S2) were annotated based on BLAST matches to known proteins in the NR database and were assigned to GO classes representing 39 terms, including some (10) that contain important information related to the enriched genotype (Figure 3).

Genes responsible for disease resistance, corresponding to the categories ‘signaling,’ ‘response to stimulus,’ ‘cellular response to stimulus,’ ‘response to chemical stimulus’ and ‘response to auxin stimulus’, were enriched in the SP81-3250, SP80-3280 and IACSP93-3046 genotypes, with IACSP93-3046 being represented in all of these categories (Figure 3). These three genotypes exhibit resistance to rust [25]–[27], whereas the other genotypes, RB925345, RB835486 and IACSP95-3018, are susceptible to rust [24], [28]. Common sugarcane rust, caused by the fungus *Puccinia melanocephaIa*, is a disease that occurs worldwide and can result in large losses of sugar tonnage in susceptible varieties [50]. Rust resistance is generally considered to be a quantitatively inherited trait showing a high degree of heritability and a strong additive genetic variance component [51], [52].

The obtained enriched terms suggest that these three genotypes harbor transcripts that are involved in stimulus response pathways and probable disease responses. These results are correlated with the characteristics of resistance and susceptibility in these varieties.

Another important characteristic of sugarcane crops is their accumulation of sucrose. Wild sugarcane species produce less than 4% fresh weight of sucrose, whereas high-yield varieties can produce sucrose contents of up to 20% of their fresh weight [53]. The major differences between these varieties is based on sugar transport and metabolism in storage tissues [54]. The entire network involving sucrose synthesis, accumulation, storage and retention is a complex system in which several metabolic pathways interact with each other [55]. The most important aspect of this network is transport, which chiefly involves specific carrier molecules, ion transport and active transport and depends on the amount of available ATP. Within this context, we observed some genotypes that were enriched in categories related to this network, particularly the transport process. These categories included ‘organic substance transport’ (SP81-3250, RB925345, SP80-3280, IACSP96-3046 and IACSP95-3018), ‘substrate-specific transporter activity’, ‘substrate-specific transmembrane transporter activity’ (SP81-3250 and SP80-3280), ‘ion transmembrane transport’ (SP81-3250 and IACSP93-3046) and ‘transporter activity’ (SP81-3250, SP80-3280, and IACSP93-3046).

Important categories involved in sugar transport and metabolism in storage tissues include the ‘monosaccharide metabolic process,’ ‘glucose metabolic process,’ ‘small molecule biosynthetic process’ and ‘small molecule metabolic process’ categories. The terms in the first and second categories were only enriched in the SP81-3250 genotype, whereas the terms in the third category were enriched in both the IACSP93-3046 and IACSP95-3018 genotypes. All genotypes showed enrichment in the last category, although SP80-3280 was the least represented.

All of the genotypes were enriched for transcripts involved in this complex network of sucrose synthesis, accumulation, storage and retention, and these results were corroborated by the agronomic characteristics of the plants. All of these genotypes produce high levels of sucrose, in accordance with the agronomic description of the genotypes SP81-3250 [26], RB925345, RB835486 [28], SP80-3280 [27], IACSP93-3046 [25] and IACSP95-3018 [24].

### Putative lncRNAs

**Figure 4:**
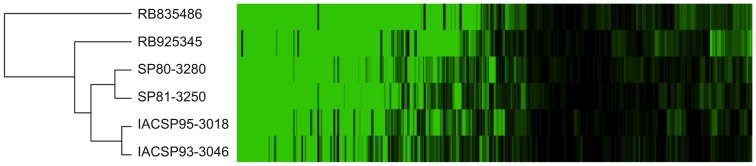
Hierarchical clustering of the 358 putative sugarcane lncRNAs. The expression patterns allowed the identification of the genotypes based on their ability to store sucrose and according to the bi-parental crosses involved in the different mapping populations.

Among the initial set of 121,342 EST retrieved unigenes, 23,529 showed no similarity to any known plant protein. These unigenes were mapped to the *S. bicolor* genome, resulting in 4,476 positive hits, with only 1,884 not exhibiting an ORF or presenting an ORF shorter than 100 aa. This subset comprised the putative sugarcane lncRNAs that are publicly available. We found that for ∼4% of these sequence, there were small RNAs (sRNAs) that mapped to their sequence, with ∼59% showing similarity to *S. bicolor* and ∼39% showing a highly stable secondary structure. In total, 1,446 non-redundant putative lncRNAs were identified that showed indirect evidence of functionality (Figure S1). We then compared this inclusive set (1,884 sequences) with the 18,910 assembled transcripts that lacked similarity to plant proteins. We observed 358 putative lncRNAs represented among the assembled transcripts, with ∼42% of these sequences showing a highly stable secondary structure and ∼40% showing evidence of transcription in the *S. bicolor* EST dataset. None of the unigenes to which sRNAs were mapped were similar to any assembled transcript. Finally, we compared the expression profiles of the putative lncRNAs between the different genotypes, which suggested that these transcripts may display genotype-specific expression patterns, as shown in Figure 4. A hierarchical clustering analysis revealed a pattern of separation between the genotypes from the different breeding programs, a result that is in accordance with the observation that the varieties from the same breeding program have the same genetic basis. We observed that the plant lncRNAs may display elevated intraspecific variation in expression, and several recent works have demonstrated that these transcripts exhibit tissue- and cell-specific expression patterns [56]–[59]. This study adds information regarding the dynamic involvement of these transcripts and reveals putative targets for further investigation [60], [61].

### Marker discovery

#### SSR discovery

Expressed sequence tag/simple sequence repeat (EST-SSR) markers are well established as important tools for researchers assessing genetic diversity and are useful in the development of genetic maps, comparative genomics and MAS breeding. Thus, the unigene sequences were searched for repeat motifs to explore the SSR profiles in the sugarcane transcriptome. A total of 5,106 SSRs were obtained from 4,616 unigene sequences (7.96%), and 576 of the unigenes contained more than one SSR (Text S7). Of these unigenes, 189 exhibited compound SSR formation. Trinucleotide repeat motifs were the most abundant, accounting for 2,585 SSRs (50.63%) in 2,318 unigene sequences; dinucleotide repeat motifs accounted for 1,927 SSRs (37.74%) in 1,732 unigenes; and other motifs accounted for 594 SSRs (11.63%) in 1,708 unigenes (Table 4). The relative percentage of the sequences containing SSRs was higher than that obtained in the SUCEST (Sugarcane Expressed Sequence Tag database) study, in which 2,005 clusters containing SSRs were found among 43,141 clusters (4.64%) [62].

**Table 4:** Summary of the simple sequence repeat (SSR) types in the sugarcane transcriptome.

Repeat motif	Number^a^	Unigenes^b^	Percentage (%)^c^
**Di-nucleotide**			
AC/GT	551		
AG/CT	962		
AT/TA	336		
CG/GC	78		
**Total**	**1,927**	**1,732**	**37.74**
**Tri-nucleotide**			
AAC/GTT	141		
AAG/CTT	152		
AAT/ATT	60		
AGC/GCT	219		
ACG/CGT	197		
AGT/ACT	62		
ACC/GGT	122		
AGG/CCT	252		
ACA/TGT	97		
AGA/TCT	46		
ATA/TAT	24		
ATC/GAT	42		
ATG/CAT	43		
CAC/GTG	69		
CAG/CTG	228		
CCG/CGG	442		
CGC/GCG	241		
CTC/GAG	148		
**Total**	**2,585**	**2,318**	**50.63**
**Other motifs** d	**594**	**1,708**	**11.63%**
**Total**	**5,106**	**5,758**	**-**

a Number of the total SSRs (di-, tri- and other motifs).

b Number of unigene sequences containing SSRs.

c The relative percentage of SSRs with different repeat motifs among the total SSRs.

d The total number of SSRs of other sizes.

The most abundant motifs included the dinucleotide AG motif (49.9%) and the trinucleotide CCG (17%) and ACC (4.7%) motifs. These results are similar to those of the SSR motif analysis performed in sorghum [63]. Additionally, CCG and ACC were the most commonly found motifs in the SUCEST study [62], and CCG was the motif that was identified most often by Cordeiro *et al*. [64]. The most frequent tetranucleotide motif found in the present study was AAAG. The overall frequency of SSRs was observed to be 1/1.6 kb.

The prevalence of trimeric motifs over other SSR repeats may be explained based on the risk of frameshift mutations that may occur when microsatellites alternate in size [65]. Furthermore, a large number of trinucleotide coding repeats appear to be controlled primarily by mutation pressure.

The development of SSR markers associated with important agronomic traits can be used to assist in the selection of varieties during the early stages of MAS breeding programs and can be helpful in the selection of the best parents for crossing [66]. Consequently, the application of such markers supports breeding programs by significantly reducing the time and cost involved in developing new varieties and can help bypass barriers in sugarcane breeding programs.

#### SNP discovery

A total of 708,125 putative SNP positions were identified (Text S5), with a density of 1 SNP per 86 bp. The frequency of SNPs found in the sugarcane genes was higher than has been observed in other grasses, such as rice and sorghum, which exhibit a frequency of ≥1 SNP per 300 bp [67]. The observed number of transitions was 456,666, and 254,658 transversions were detected, with the number of the former being 1.79 times that of the latter. Transitions were most likely more frequent because they are more tolerated by natural selection as the tendency to generate synonymous mutations in coding sequences is related to the number of transversions [68].

We identified SNPs in 58,903 different unigenes, which represent 81.50% of the total unigenes. Considering the number of unigenes without SNPs, we verified that 10,516 (79%) are unigenes with a length of less than 500 bp. Considering only those unigenes with predicted ORFs (33,673 unigenes), we found a total of 289,969 SNPs (37.5% of the total detected SNPs).

**Figure 5:**
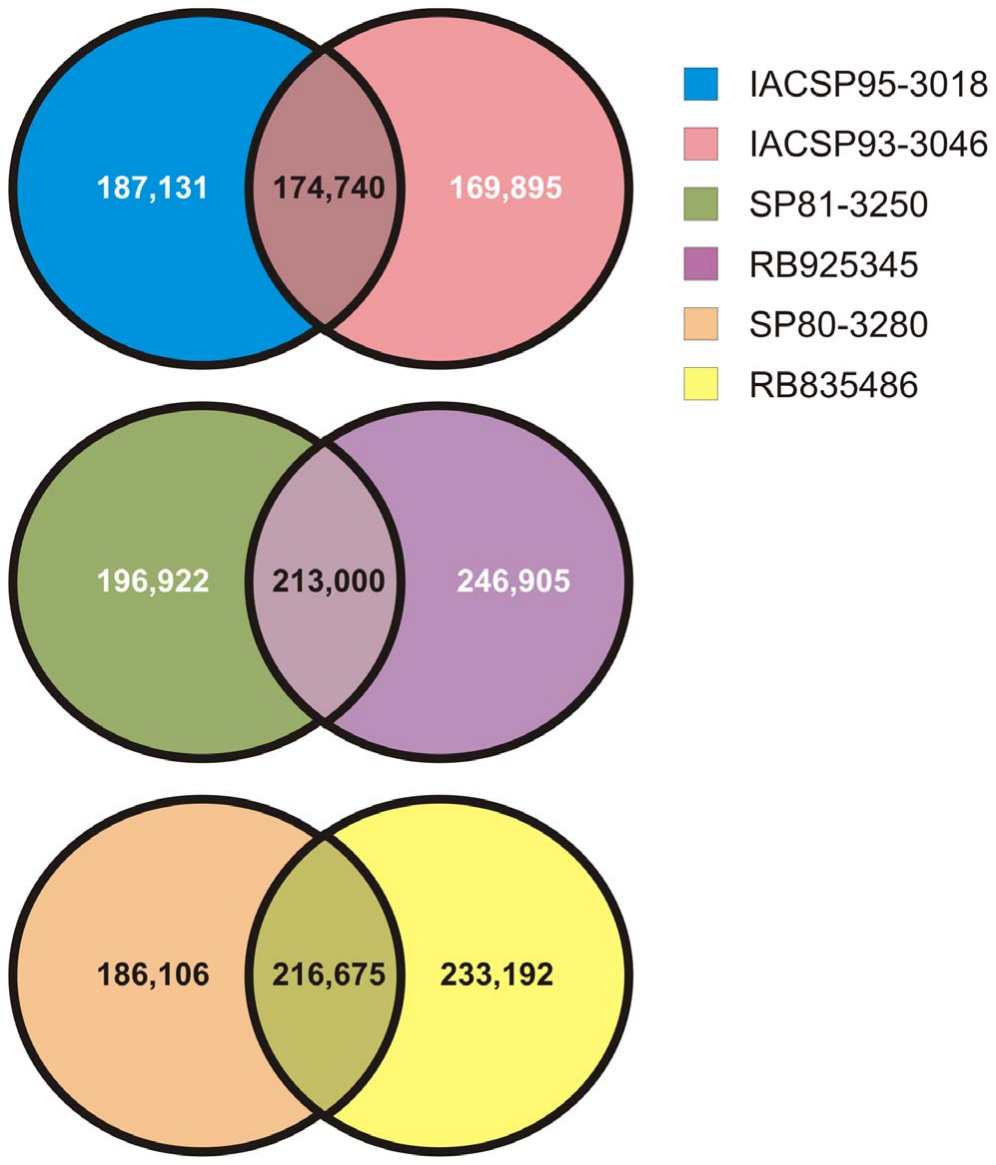
Unique and shared heterozygous putative SNPs in the parental genotypes of the three sugarcane mapping populations.

To detect different heterozygous SNPs between the parents from each mapping population, the reads from each genotype were mapped against all the unigenes (Text S6). Figure 5 shows the heterozygous SNPs that were detected, and the unique and shared SNPs in each parent from the mapping populations were evaluated. The percentages of SNPs that were common in the three mapping populations, IACSP95-3018×IACSP93-3046 (32.86%), SP81-3250×RB925345 (32.42%) and SP80-3280×RB835486 (34.06%), were similar, and these SNPs may thus be polymorphic between the parents. As sugarcane is a polyploid species, polymorphisms can be generated from a different number of allelic copies present in each genotype. However, such polymorphisms are difficult to validate (Garcia *et al* 2013, *submitted*).

The SNPs that were unique to each genotype (Figure 5) exhibited a higher probability of association with the contrasting agronomic traits of interest. Because polymorphism markers between parents are important for generating saturated genetic mapping in mapping populations, these SNPs are a source of data for generating markers associated with quantitative trait loci (QTLs). Such functional molecular markers have been broadly applied for the genetic improvement of several crops [69].

According to the Gene Ontology annotation, we identified SNPs in 6,712 unigenes with annotation information, representing 44.80% of the unigenes included in the enrichment analyses. Some categories exhibited important results related to the genotype (Figure 3), particularly those associated with disease resistance. In the ‘signaling’ category, we identified 161 unigene sequences with SNPs, whereas we identified 477 unigenes with SNPs in the ‘response to stimulus’ category. These unigenes likely represent source data for the development of functional markers related to disease resistance.

When we analyzed the categories related to sucrose synthesis, accumulation, storage and retention, we also observed unigenes with SNPs in the ‘organic substance transport’ (226), ‘substrate-specific transporter activity’ (196) and ‘ion transmembrane transport’ (53) clusters. Equally important categories involving sugar transport and metabolism in storage tissues, such as the ‘glucose metabolic process’ (43), ‘small molecule biosynthetic process’ (133) and ‘small molecule metabolic process’ (414) categories, also containing unigene sequences with SNPs.

All of these unigene sequences with SNPs represent an important source of data. These sequences could be priority candidates for the development of specific functional markers and could be very useful in further genetic or genomic studies in sugarcane.

## Conclusion

This is the first publicly available sugarcane transcriptome sequencing study performed using NGS technology to investigate the entire sugarcane transcriptome, and our data provide the most comprehensive transcriptome resource currently available for sugarcane. In addition, polymorphisms associated with candidate genes potentially involved in the stimulus response, energy production and growth were identified among the contrasting varieties and deserve future investigation. Based on the enrichment analysis, we identified putative genes related to disease and the accumulation of sucrose. Additionally, a large number of SNPs and SSRs were identified, and marker development would be a useful resource for future genetic or genomic studies of this species. Finally, this work contributed information on 5,000 undescribed genes, which is more than half of the expected sugarcane genes that are missing from sugarcane databases.

## Supporting Information

Figure S1 Venn diagram showing the classification of the identified putative sugarcane lncRNAs in the EST data (A) and RNA-Seq data (B).
(TIF)

Text S1 Unigene sequences in FASTA format.
(ZIP)

Text S2 Gene ontology enrichment annotation for the transcripts of each genotype.
(ZIP)

Text S3 Putative previously unknown sugarcane transcripts showing the best matches to sorghum proteins.
(TXT)

Text S4 List of 18,910 putative sugarcane ncRNAs with high coverage in the sorghum genome.
(TXT)

Text S5 List of 708,125 putative SNP positions identified in this study.
(ZIP)

Text S6 List of putative SNPs identified in each genotype.
(ZIP)

Text S7 List of 5,106 putative SSR positions identified in this study.
(XLS)
