# Supplementary figures and images for: De Novo Assembly and Transcriptome Analysis of Contrasting Sugarcane Varieties
Source: PLoS One. 2014 Feb 11;9(2):e88462. doi: 10.1371/journal.pone.0088462 (PMC3921171; doi:10.1371/journal.pone.0088462)

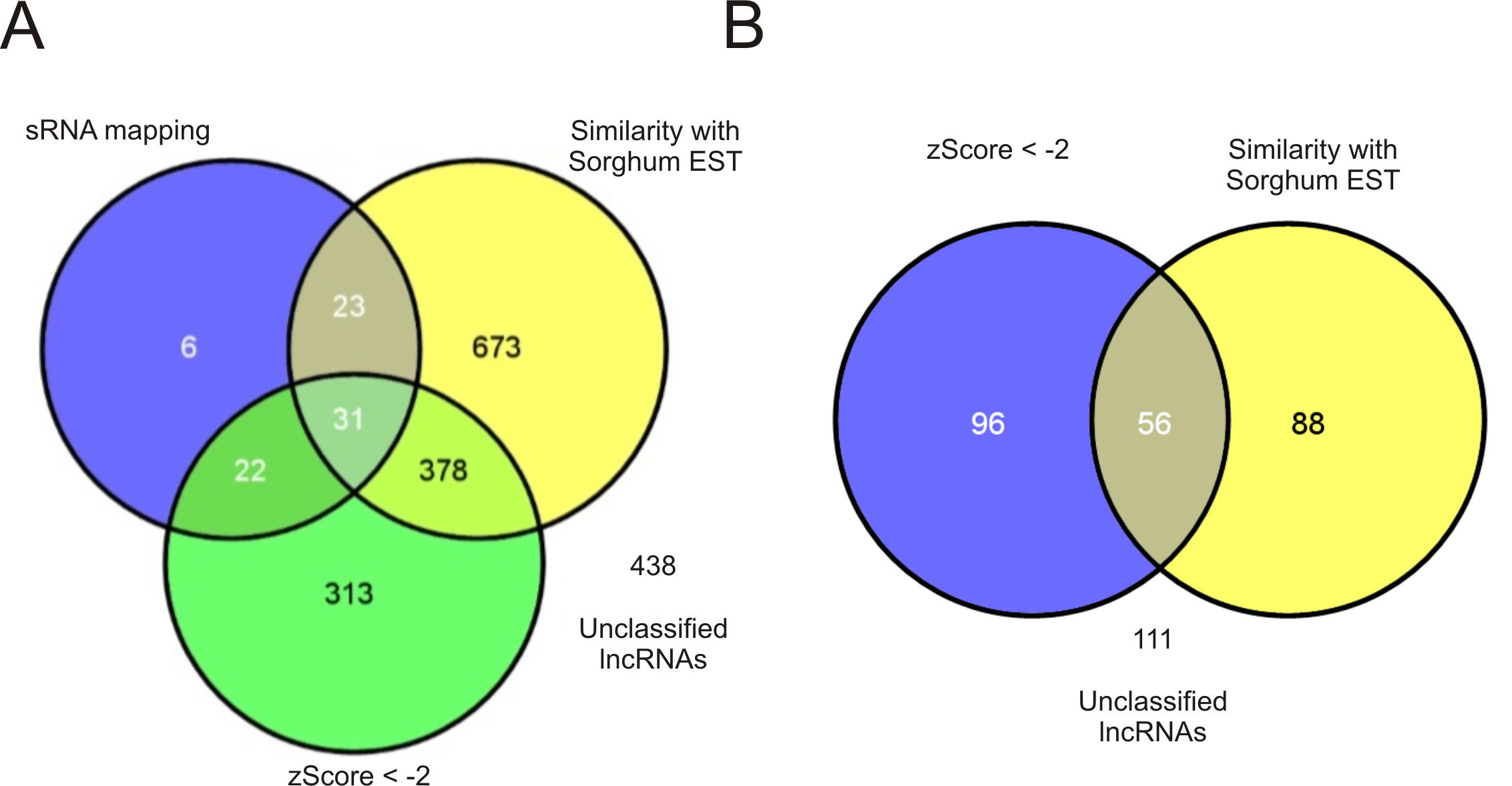

Supplement: Figure S1 — Venn diagram showing the classification of the identified putative sugarcane lncRNAs in the EST data (A) and RNA-Seq data (B). (TIF) [file pone.0088462.s001.tif]
